# Clinical Outcomes for PD-1 Inhibitor Plus Chemotherapy as Second-Line or Later Therapy Compared to PD-1/PD-L1 Inhibitor Alone in Advanced Non-small-cell Lung Cancer

**DOI:** 10.3389/fonc.2020.556275

**Published:** 2020-09-30

**Authors:** Xiaoyang Zhai, Xuquan Jing, Ji Li, Yaru Tian, Shuhui Xu, Min Wang, Hui Zhu

**Affiliations:** ^1^Department of Radiation Oncology, Shandong Cancer Hospital and Institute, Shandong First Medical University and Shandong Academy of Medical Sciences, Jinan, China; ^2^Department of Radiation Oncology, Shandong Cancer Hospital and Institute Affiliated of Shandong University, Jinan, China

**Keywords:** non-small-cell lung cancer, PD-1, PD-L1, second-line or later therapy, immune-related adverse events

## Abstract

**Background:**

Programmed death-1 (PD-1)/programmed death-ligand 1 (PD-L1) inhibitor monotherapy has been approved as second-line or later therapy in advanced non-small-cell lung cancer (NSCLC). The study aimed to compare the clinical outcomes of PD-1 inhibitor plus chemotherapy with PD-1/PD-L1 inhibitor monotherapy as second-line or later therapy in advanced NSCLC.

**Methods:**

The clinical data of patients with advanced NSCLC who received PD-1/PD-L1 inhibitors as second-line or later line therapy was retrospectively collected. Patients were assigned to one of the two groups according to the therapeutic modality used: PD-1/PD-L1 inhibitor monotherapy group or PD-1 inhibitor plus chemotherapy combination therapy group. Disease control rate (DCR), progression-free survival (PFS), and overall survival (OS) were evaluated between the two groups. The prognostic effect of the derived neutrophil-to-lymphocyte ratio (dNLR) and lactate dehydrogenase (LDH) on the outcomes was also evaluated.

**Results:**

From April 2017 to October 2019, a total of 84 patients were enrolled in the current study. Twenty-six patients (PD-1 inhibitor, *n* = 25; PD-L1 inhibitor, *n* = 1) received PD-1/PD-L1 inhibitor monotherapy, and fifty-eight patients received PD-1 inhibitor plus chemotherapy. The chemotherapy regimens used were as follows: liposome paclitaxel (*n* = 15); nab-paclitaxel (*n* = 12); docetaxel (*n* = 9); pemetrexed (*n* = 6); and others (*n* = 16). The DCR and OS were not significantly different between the two groups. The PFS of the monotherapy group was longer than that of the combination therapy group (mPFS: 9.6 vs. 4.6 months, *P* = 0.01). Univariate and multivariate analyses suggested that LDH and sex were independent prognostic factors of PFS. In the second-line therapy subgroup of 38 patients, OS and PFS were not significantly different between the two groups. In the subgroup of 46 patients treated beyond the 2nd line, the monotherapy group had a longer PFS (mPFS: 9.6 vs. 4.2 months, *P* = 0.01). The incidence of any-grade adverse events was not significantly different between the monotherapy group and the combination therapy group (19.2 vs. 18.9%, *P* = 1.000). One patient in the PD-1 inhibitor plus chemotherapy group died of immune-related pneumonitis.

**Conclusion:**

The clinical outcomes of PD-1 inhibitor plus chemotherapy as second-line or later therapy were similar to those of PD-1/PD-L1 inhibitor alone in advanced NSCLC.

## Introduction

Lung cancer is still the malignant tumor with the highest incidence and mortality worldwide (1). Non-small-cell lung cancer (NSCLC) accounts for approximately 80 to 85% of all lung cancers (2). Despite recent advances in treatment, advanced NSCLC still has a poor prognosis. Especially for patients whose disease progresses after first-line therapy, alternative drugs are limited. Docetaxel is used as the second- and third-line therapy, with a median overall survival of only 7.5 months and survival rates of 32% at 1 year (3, 4). The antibodies that target programmed death-1 (PD-1) and programmed death-ligand 1 (PD-L1) have been approved for second-line or subsequent therapy, showing overall survival (OS) benefits over docetaxel.

The approval of these PD-1/PD-L1 antibodies was mainly based on the results of the CheckMate 017, CheckMate 057, KEYNOTE 010 and OAK trials (5–8). In these trials, nivolumab, used as second-line therapy, showed a 5-year survival rate above fourfold higher than docetaxel (13 vs. 3%, hazard ratio 0.68, 95% CI 0.59–0.78) (9). Compared with docetaxel, pembrolizumab showed a median OS of 10.4 vs. 8.5 months (HR 0.71, 95% CI 0.58–0.88) at the approval dose of 2 mg/kg in NSCLC patients whose PD-L1 expression is 1% or above in tumor cells (7). Moreover, atezolizumab also showed longer OS (13.8 vs. 9.6 months, HR 0.73, 95% CI 0.62–0.87) than docetaxel in patients with previously treated advanced NSCLC (8).

Chemotherapy, as a conventional therapy mode, has a synergistic antitumor effect with the PD-1/PD-L1 inhibitor. Chemotherapy can enhance the immune response regained by PD-1/PD-L1 inhibitors by a series of underlying mechanisms, such as strengthening the sensitivity of tumor cells to the lysis effect of cytotoxic T lymphocytes (CTLs) (10), increasing the immunogenicity of tumor cells (11), reducing immunosuppressive cells by inducing cell apoptosis (12, 13), and promoting the antitumor immune response by changing the tumor microenvironment (14). PD-1/PD-L1 inhibitors combined with chemotherapy, approved as a first-line therapy, has given more benefit to extensive population (15, 16). However, the efficacy and safety of PD-1/PD-L1 inhibitors plus chemotherapy as second-line or later therapy remained unclear. Therefore, we performed this retrospective study to explore the clinical efficacy and safety of second-line or later therapy with PD-1/PD-L1 inhibitors plus chemotherapy in advanced NSCLC patients and to compare the clinical outcomes of PD-1/PD-L1 inhibitors plus chemotherapy with PD-1/PD-L1 inhibitor monotherapy as second-line or later therapy further.

## Materials and Methods

### Patients

We retrospectively reviewed the records of advanced NSCLC patients who received a PD-1/PD-L1 inhibitor as second-line or later therapy in Shandong Cancer Hospital and Institute (Jinan, Shandong, China) between April 2017 and October 2019. Eligible patients were histologically or cytologically diagnosed with NSCLC, had a Karnofsky Performance Status (KPS) score ≥80, and had measurable lesions. Patients harboring epidermal growth factor receptor (EGFR) mutations or anaplastic lymphoma kinase (ALK) fusions and those who suffered from failure of tyrosine kinase inhibitor (TKI) therapy were also enrolled in the study. Patients with autoimmune disease, interstitial lung disease and immunosuppression and those who were previously treated with a cytotoxic T-lymphocyte antigen-4 (CTLA-4) inhibitor, PD-1/PD-L1 inhibitor or immunosuppressant were excluded. Patients without response evaluation because of fewer than 2 cycles or loss to follow-up were also excluded. The study was approved by the Ethics Committee of Shandong Cancer Hospital and Institute. All procedures involving patients conformed to the principles outlined in the Declaration of Helsinki.

### Treatments

The patients were divided into the PD-1/PD-L1 inhibitor monotherapy group and the PD-1 inhibitor plus chemotherapy combination therapy group based on their treatment modality. The PD-1/PD-L1 inhibitors in the monotherapy group included sintilimab, pembrolizumab, nivolumab, camrelizumab, atezolizumab and RB004. The PD-1 inhibitors in the PD-1 inhibitor plus chemotherapy group included sintilimab, pembrolizumab, nivolumab, camrelizumab, and toripalimab. The chemotherapy regimens involved liposome paclitaxel, nab-paclitaxel, docetaxel, pemetrexed, and others. The PD-1/PD-L1 inhibitors and chemotherapy drugs were all administered intravenously. The therapeutic schedule for each patient was decided by the attending physicians based on the efficacy of the previous therapy and the patient’s physical condition and intentions.

### Assessment of Response and Toxicity

The response evaluation of tumors was based on the Response Evaluation Criteria in Solid Tumors (RECIST) version 1.1. Evaluation was performed routinely every 6–8 weeks after starting treatment with the PD-1/PD-L1 inhibitor. Adverse events (AEs) were assessed according to the National Cancer Institute Common Terminology Criteria for Adverse Events (CTCAE) version 4.0. AEs that occurred during hospitalization were registered and graded by their attending doctor timely, while AEs that occurred outside the hospital were mainly based on patients’ initiative report.

### Endpoints

The primary endpoint was OS, which was defined as the time interval from the treatment initiation of the PD-1/PD-L1 inhibitor to death caused by any reason or the last known follow-up. The secondary endpoints were progression-free survival (PFS), disease control rate (DCR), and AEs. PFS was measured as the time interval from the initiation of PD-1/PD-L1 inhibitor treatment to tumor progression, death from any cause or the last known follow-up. According to RECIST version 1.1, objective response rate (ORR) refers to the proportion of patients who had a complete or partial response, and the DCR refers to that of patients who had a complete or partial response or stable disease (SD). Moreover, we also collected the pretreatment (within 30 days before the first PD-1/PD-L1 inhibitor treatment)-derived neutrophil to lymphocyte ratio (dNLR) and lactate dehydrogenase (LDH) to explore their prognostic effects on the outcome of advanced NSCLC patients. The dNLR is calculated by neutrophils/leukocytes minus neutrophils. dNLR > 3 and LDH > upper limit of normal (ULN) were defined as high (17, 18).

### Statistical Analysis

All statistical analyses were performed using GraphPad Prism software version 8.0 (GraphPad Software, Inc., United States) and SPSS statistical software version 20.0 (IBM Corp., United States). The comparisons of patients’ baseline characteristics, tumor response and AEs in the two groups were analyzed using the Chi-square test and Fisher’s exact test. Univariate survival analysis was performed using the Kaplan–Meier method. Multivariate survival analysis was performed by a Cox proportional hazards model to evaluate the independent prognostic factors associated with improved survival. The Kaplan–Meier method was used to calculate OS and PFS. The difference in survival curves between the two groups was estimated by the log-rank test. Two-sided *P* values < 0.05 were considered statistically significant.

## Results

### Patient Characteristics

**FIGURE 1:**
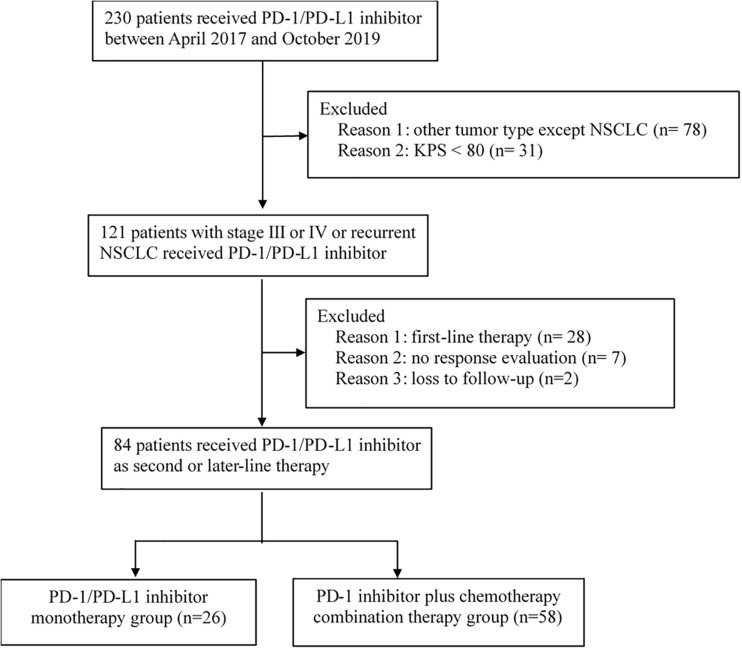
Study diagram.

Between April 2017 and October 2019, 230 patients received immunotherapy in our cancer center. 78 patients were excluded for other tumor types, and 31 patients were excluded for KPS < 80. The total number of patients with stage IIIB or IV or recurrent NSCLC who received PD-1/PD-L1 inhibitor treatment was 121. Twenty-eight patients receiving PD-1/PD-L1 inhibitor as first-line therapy and seven patients without response evaluation were excluded. The follow-up rate was 97.6%, and two patients were lost to follow-up (Figure 1). As a result, a total of 84 patients who received PD-1/PD-L1 inhibitor as the second-line or later therapy were enrolled in the study. According to the therapeutic modality, there were 26 patients in the monotherapy group and 58 patients in the combination group. A total of 62.1% of patients in the combination group received taxane drugs, including liposome paclitaxel, nab-paclitaxel, and docetaxel. The last follow-up date was June 30, 2020. Fifty-three patients were still alive and thirty-one patients were died by the end of the follow-up. The median follow-up time was 11.4 months (range, 2.1–24.7 months) for all patients and 12.6 months (range, 8.8–24.7 months) for living patients. The baseline characteristics of all patients in the two groups are presented in Table 1. There were no differences in the distribution of most variables other than histologic features between the two groups. The rate of adenocarcinoma patients was 74.1% in the combination group and 42.3% in the monotherapy therapy group. The median age of the patients in the two groups was 62 years, and the age range was 29 to 81 years. Thirty-eight (45.2%) patients had received second-line therapy, and 46 (54.8%) patients had received third-line or later therapy. Twenty (23.8%) patients had developed brain metastases, and 13 (15.4%) patients had developed liver metastases. Forty-five (53.5%) patients were never smokers, which is higher than that reported in clinical trials. Moreover, in our study, there were 9 patients (10.7%) with tumors harboring EGFR mutations and 2 patients with tumors harboring ALK fusions.

**TABLE 1:** Clinical features of patients with advanced NSCLC receiving second-line or later therapy in the monotherapy group or the combination therapy group.

**Characteristic**	**Total**	**Monotherapy group**		**Combination therapy group**		***P***
		**No.**	**%**	**No.**	**%**	
**Gender**						
Male	60	22	84.6	38	65.5	
Female	24	4	15.4	20	34.5	0.073
**Age (years)**						
Range	29–81	43–81		29–79		
Median	62	60		62		
<65	57	17	65.4	40	69.0	
≥65	27	9	34.6	18	31.0	0.745
**KPS score**						
≥90	38	12	46.2	26	44.8	
80–90	46	14	53.8	32	55.2	0.910
**Smoking status**						
Current or former smoker	39	13	50.0	26	44.8	
Never smoker	45	13	50.0	32	55.2	0.660
**Histologic features**						
Adenocarcinoma	54	11	42.3	43	74.1	
Squamous	25	13	50.0	12	20.7	
Other	5	2	7.7	3	5.2	0.011
**Line of therapy**						
Second	38	14	53.8	24	41.4	
Third or later	46	12	46.2	34	58.6	0.289
**Brain metastases**						
Yes	20	4	15.4	16	27.6	
No	64	22	86.6	42	72.4	0.225
**Liver metastases**						
Yes	13	1	3.8	12	20.7	
No	71	25	96.2	46	79.3	0.056
**Previous radiotherapy**						
Yes	49	13	50.0	36	62.1	
Thoracic radiotherapy	30	10		20		
Other or unknown	19	3		16		
No	35	13	50.0	22	37.9	0.300
**EGFR mutation**						
Yes	9	2	7.7	7	12.1	
No	51	13	50.0	38	65.5	
Unknown	24	11	42.3	13	22.4	0.172
**PD-L1 expression**						
Positive	16	7	26.9	9	15.5	
Negative	8	2	7.7	6	10.3	
Unknown	60	17	65.4	43	74.1	0.481
**LDH level**^ 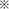 ^	82	24		58		
>ULN	34	7	29.2	27	46.6	
≤ULN	48	17	70.8	31	53.4	0.146
**dNLR level**^ 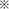 ^	82	24		58		
>3	23	6	25.0	17	29.3	
≤3	59	18	75.0	41	70.7	0.693
**Immunotherapy drugs**						
Sintilimab	45	10	38.5	35	60.3	
Pembrolizumab	15	5	19.2	10	17.2	
Nivolumab	10	5	19.2	5	8.6	
Other checkpoint inhibitors	14	6	22.1	8	13.8	0.204

*^
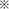
^Two patients with unknown dNLR and LDH were unevaluable. NSCLC, non-small-cell lung cancer; KPS, Karnofsky performance status; AJCC, American Joint Committee on Cancer; EGFR, epidermal growth factor receptor; LDH, lactate dehydrogenase; ULN, upper limit of normal; dNLR, derived neutrophil to lymphocyte ratio; *χ2*, Chi-square statistic.*

Among the 84 patients, 24 patients had information available regarding the PD-L1 tumor proportion score (TPS). 33.3% (8/24) patients had PD-L1 TPS scores that were less than 1%, 25% (6/24) had TPS scores between 1 and 50%, and 41.6% (10/24) had TPS scores greater than or equal to 50%. Positive rates of tumor PD-L1 expression were balanced between the monotherapy group and the combination therapy group (Table 1).

### Response

Among all 84 patients, the ORR was 16.7%, and the DCR was 76.2%. The ORR was 19.2% in the monotherapy group and 15.5% in the combination therapy group (*P* = 0.832). The DCR was 80.8% in the monotherapy group and 74.1% in the combination therapy group (*P* = 0.509). The difference in the response rate between these two groups was not significant (Table 2).

**TABLE 2:** Tumor response in patients with advanced NSCLC receiving second-line or later therapy in the monotherapy group or the combination therapy group.

	**Monotherapy group (*n* = 26)**	**Combination therapy group (*n* = 58)**
**Objective response rate, n (%)**	5 (19.2%)	9 (15.5%)
*P* value	0.832	
**Disease control rate, n (%)**	21 (80.8%)	43 (74.1%)
*P* value	0.509	
**Best overall response, n (%)**		
Complete response	0	0
Partial response	5 (19.2%)	9 (15.5%)
Stable disease	16 (61.5%)	34 (58.6%)
Progressive disease	5 (19.2%)	15 (25.9%)

*PR, partial response; SD, stable disease; PD, progressive disease; DCR, disease control rate.*

### Survival

**FIGURE 2:**
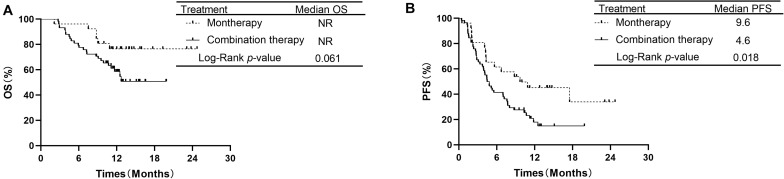
Comparison of OS and PFS of patients with advanced NSCLC receiving second-line or later therapy between PD-1/PD-L1 inhibitor alone and PD-1 inhibitor plus chemotherapy. **(A)** Comparison of OS. **(B)** Comparison of PFS. NSCLC, non-small-cell lung cancer; PD-1, programmed death-1; PD-L1, programmed death-ligand 1; OS, overall survival; PFS, progression-free survival.

The median OS in the monotherapy group and in the combination therapy group were not reached (NR). The OS rates at 1 year in the monotherapy group and combination therapy group were 76.5 and 58.6%, respectively. OS was not significantly different between the PD-1/PD-L1 monotherapy group and the PD-1 plus chemotherapy group among all 84 patients (*P* = 0.061, Figure 2A). The median PFS was 9.6 months for the patients receiving monotherapy and 4.6 months for those receiving combination therapy. PFS was longer in the PD-1/PD-L1 inhibitor monotherapy group than in the PD-1 inhibitor plus chemotherapy group (*P* = 0.018, Figure 2B). The PFS rates at 1 year in the monotherapy group and combination therapy group were 45.1 and 17.8%, respectively.

**FIGURE 3:**
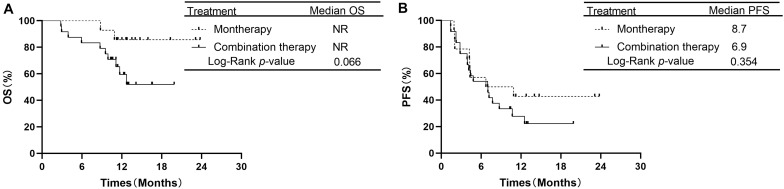
Overall survival and progression free survival in subgroup analysis of second-line therapy between PD-1/PD-L1 inhibitor alone and PD-1 inhibitor plus chemotherapy. **(A)** Comparison of OS. **(B)** Comparison of PFS. NSCLC, non-small-cell lung cancer; PD-1, programmed death-1; PD-L1, programmed death-ligand 1; OS, overall survival; PFS, progression-free survival.

**FIGURE 4:**
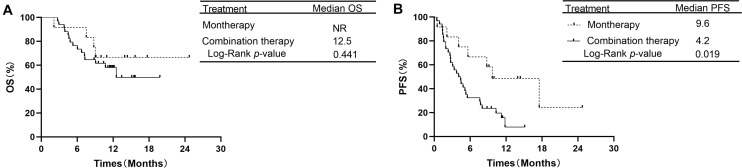
OS and PFS in subgroup analysis of treatment beyond second-line therapy between PD-1/PD-L1 inhibitor alone and PD-1 inhibitor plus chemotherapy. **(A)** Comparison of OS. **(B)** Comparison of PFS. NSCLC, non-small-cell lung cancer; PD-1, programmed death-1; PD-L1, programmed death-ligand 1; OS, overall survival; PFS, progression-free survival.

In the subgroup of 38 patients receiving second-line therapy, the OS was not significantly different between the PD-1/PD-L1 monotherapy group and the PD-1 plus chemotherapy group (median OS: NR vs. NR, *P* = 0.066, Figure 3A). The OS rates at 1 year in the monotherapy group and combination therapy group were 85.7 and 59.4%, respectively. The difference in PFS between the two groups was also not significant (median PFS: 8.7 vs. 6.9 months, *P* = 0.354, Figure 3B). In the subgroup of 46 patients receiving treatment beyond second-line therapy, patients in the monotherapy group had a longer PFS than those in the combination therapy group (median PFS: 9.6 vs. 4.2 months, *P* = 0.019, Figure 4B). The OS between the two groups was not significantly different (median OS: NR vs. 12.5 months, *P* = 0.441, Figure 4A).

**FIGURE 5:**
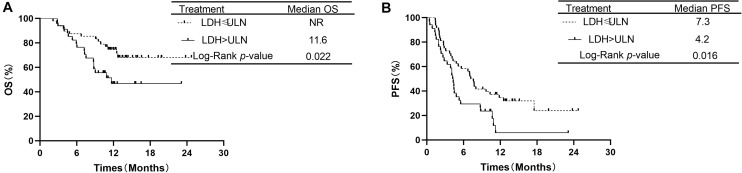
Comparison of overall survival and progression-free survival of patients with LDH ≤ ULN vs. LDH > ULN.

Additionally, the analysis showed that OS was significantly longer in patients with LDH ≤ ULN than in patients with LDH > ULN. The median OS was 11.6 months in the group of LDH > ULN and NR in group of LDH ≤ ULN (*P* = 0.022, Figure 5A). The median PFS was 4.2 months in patients of LDH > ULN and 7.3 months in patients of LDH ≤ ULN. This difference was also significant (*P* = 0.016, Figure 5B).

### Prognostic Factors

The clinical characteristics of the patients were evaluated to determine their prognostic value for OS (Table 3). Univariate analysis indicated that age, sex, KPS score, smoking status, histologic features, line of therapy, brain metastases, liver metastases, previous radiotherapy, EGFR mutation status, dNLR, immunotherapy drugs and treatment mode were not associated with survival. LDH ≤ ULN was associated with better OS than LDH > ULN (*P* = 0.022). However, multivariate analyses indicated that no clinical factors were associated with OS. For PFS, univariate analysis revealed that male sex, non-adenocarcinoma status, non-liver metastases, LDH ≤ ULN and monotherapy were significant favorable prognostic factors (Table 4). Multivariate analysis revealed that male sex (*P* = 0.035) and LDH ≤ ULN (*P* = 0.043) were favorable prognostic factors for PFS.

**TABLE 3:** Univariate analysis and multivariate analysis of the prognostic factors for OS in patients with advanced NSCLC receiving second-line or later-line therapy.

	**Univariate analysis**			**Multivariate analysis**		
**Factors**	**MST (m)**	***χ 2***	***P***	**HR**	**95% CI**	***P***
**Gender**						
Male	−					
Female	11.6	2.837	0.092	0.546	0.177–1.684	0.292
**Age (years)**						
<65	−					
≥65	−	0.065	0.798	0.693	0.300–1.604	0.392
**KPS score**						
≥90	−					
80–90	−	3.232	0.072	0.520	0.230–1.173	0.115
**Smoking status**						
Current or former smoker	−					
Never smoker		0.006	0.937	0.604	0.190–1.920	0.393
**Histologic features**						
Adenocarcinoma						
Non-adenocarcinoma	−	1.179	0.278	1.117	0.434–2.877	0.819
**Line of therapy**						
Second	−					
Third or later		1.569	0.210	0.707	0.314–1.592	0.402
**Brain metastases**						
Yes	−					
No	−	0.887	0.346	0.918	0.350–2.404	0.861
**Liver metastases**						
Yes	10.7					
No	−	3.507	0.061	0.875	0.353–2.171	0.774
**Previous radiotherapy**						
Yes	−					
No	−	0.003	0.958	0.879	0.407–1.900	0.744
**EGFR mutation^ 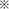 ^**						
Yes	10.7					
No	−	1.538	0.215			
**LDH**						
>ULN	11.6					
≤ULN	−	5.220	0.022	2.171	0.981–4.808	0.056
**dNLR**						
>3	−					
≤3	−	1.271	0.260	1.152	0.493–2.692	0.744
**Immunotherapy drugs**						
Sintilimab	−					
Pembrolizumab	−					
Nivolumab	−					
Other checkpoint inhibitors	10.8	2.982	0.394			
**Treatment mode**						
Monotherapy	−					
Combination	−	3.491	0.062	0.686	0.250–1.881	0.464

*^
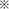
^Indicates that the patients’ EGFR mutation status was assessable. NSCLC, non-small-cell lung cancer; MST, median survival time; KPS, Karnofsky performance status; AJCC, American Joint Committee on Cancer; EGFR, epidermal growth factor receptor; LDH, lactate dehydrogenase; ULN, upper limit of normal; dNLR, derived neutrophil to lymphocyte ratio; *χ2*, Chi-square statistic.*

**TABLE 4:** Univariate analysis and multivariate analysis of the prognostic factors for PFS in patients with advanced NSCLC receiving second-line or later-line therapy.

	**Univariate analysis**			**Multivariate analysis**		
**Factors**	**Median, m**	***χ 2***	***P***	**HR**	**95% CI**	***P***
**Gender**						
Male	7.1					
Female	3.0	6.784	0.009	0.409	0.179–0.937	0.035
**Age (years)**						
<65	5.2					
≥65	7.0	1.148	0.284	1.522	0.809–2.861	0.192
**KPS score**						
≥90	5.5					
80–90	4.8	0.774	0.379	0.811	0.471–1.396	0.449
**Smoking status**						
Current or former smoker	6.9					
Never smoker	4.4	0.720	0.396	0.683	0.314–1.489	0.338
**Histologic features**						
Adenocarcinoma	4.4					
Non-adenocarcinoma	10.3	8.800	0.003	1.574	0.814–3.043	0.177
**Line of therapy**						
Second	6.9					
Third or later	4.8	1.594	0.207	0.668	0.386–1.156	0.150
**Brain metastases**						
Yes	4.0					
No	6.9	0.791	0.374	0.843	0.413–1.719	0.638
**Liver metastases**						
Yes	3.7					
No	6.7	6.016	0.014	0.716	0.354–1.448	0.353
**Previous radiotherapy**						
Yes	4.4					
No	6.7	0.000	0.993	0.792	0.444–1.410	0.428
**EGFR mutation^ 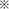 ^**						
Yes	4.0					
No	5.2	1.807	0.179			
**LDH**						
>ULN	4.2					
≤ULN	7.1	5.737	0.017	1.823	1.019–3.259	0.043
**dNLR**						
>3	4.4					
≤3	6.7	0.210	0.647	0.645	0.329–1.265	0.202
**Immunotherapy drugs**						
Sintilimab	4.4					
Pembrolizumab	4.8					
Nivolumab	6.7					
Other checkpoint inhibitors	7.0	1.394	0.707			
**Treatment mode**						
Monotherapy	9.6					
Combination therapy	4.4	5.737	0.017	0.874	0.450 –1.696	0.668

*^
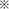
^Indicates that the patients’ EGFR mutation status was assessable. NSCLC, non-small-cell lung cancer; KPS, Karnofsky performance status; AJCC, American Joint Committee on Cancer; EGFR, epidermal growth factor receptor; LDH, lactate dehydrogenase; ULN, upper limit of normal; dNLR, derived neutrophil to lymphocyte ratio; *χ2*, Chi-square statistic.*

### Toxicities

The incidence of AEs is shown in Table 5. The rate of any grade AEs was 19.2% (5/26) in the PD-1 inhibitor monotherapy group and 18.9% (11/58) in the PD-1 plus chemotherapy group. The rate of grade 3-4 AEs was relatively higher in the combination group than in the monotherapy group (10.2 vs. 7.6%, *P* = 1.000). However, the difference was not significant. The most common grade 3–4 AEs were pneumonitis in the monotherapy group and pneumonitis and myocarditis in the combination group. One patient (3.8%) in the monotherapy group withdrew treatment because of pneumonitis, and 5 patients (8.5%) in the combination group discontinued treatment because of pneumonitis, myocarditis, and diarrhea. One patient in the combination group died of immune-related pneumonitis.

**TABLE 5:** Incidence of adverse events (AEs).

**Treatment-related AEs, n (%)**	**Monotherapy group (*n* = 26)**	**Combination therapy group (*n* = 58)**	***P***
**Any grade**	5 (19.2%)	11 (18.9%)	1.000
Fatigue	0 (0)	1 (1.7%)	
Rash	1 (3.8%)	1 (1.7%)	
Diarrhea	1 (3.8%)	2 (3.4%)	
Decreased weight	1 (3.8%)	0	
Decreased appetite	0	1 (1.7%)	
Myocarditis	0	4 (6.8%)	
Pneumonitis	2 (7.6%)	4 (6.8%)	
Hypothyroidism	0	1 (1.7%)	
Neutrophil count decreased	0	3 (5.1%)	
**Grade ≥ 3**	2 (7.6%)	6 (10.2%)	1.000
Neutrophil count decreased	0	1 (1.7%)	
Pneumonitis	1 (3.8%)	2 (3.4%)	
Myocarditis	0	2 (3.4%)	
Diarrhea	0	1 (1.7%)	
Decreased weight	1 (3.8%)	0	
**AEs leading to discontinuation**	1 (3.8%)	5 (8.5%)	
Pneumonitis	1 (3.8%)	2 (3.4)	
Myocarditis	0	2 (3.4)	
Diarrhea	0	1 (1.7%)	
**AEs leading to death**	0	1 (1.7%)	
Pneumonitis	0	1 (1.7%)	

*AE, adverse event.*

## Discussion

Data from KEYNOTE 189, KEYNOTE 407, and the IMPOWER 150 trial indicated that chemotherapy combined with immunotherapy (15, 19, 20) had superior efficacy over conventional chemotherapy as a first-line therapy for advanced NSCLC, with median survival times of 22, 15.9, and 19.2 months (19–21), respectively. Moreover, as second-line or later therapy, data from CheckMate 017 and CheckMate 057 (5, 6) also revealed that the PD-1/PD-L1 inhibitor alone had superior efficacy over docetaxel. However, whether chemotherapy plus PD-1/PD-L1 inhibitor as a second-line or later therapy is superior to PD-1/PD-L1 inhibitor alone remained unclear. Our study revealed that addition of chemotherapy to PD-1 inhibitors did not improve OS or PFS compared with PD-1/PD-L1 inhibitor alone for NSCLC patients as second-line and later therapy. PFS in the monotherapy group was longer than that in the PD-1 inhibitor plus chemotherapy group. Univariate and multivariate analyses suggested that male sex and LDH ≤ ULN were independent favorable factors for PFS. The incidence of grade 3–4 AEs in the combination therapy group was relatively higher than that in the monotherapy group, although there was no significant difference between the two groups. In the current study, one patient died of immune-related pneumonitis.

Notably, our study found that combination therapy group was superior to monotherapy group in the early stage of OS and PFS curves. In early stage of the survival curves of CheckMate 057, nivolumab was inferior to docetaxel (6). The analysis of early survival in CheckMate 057 indicated that death within the first three months of treatment was mainly due to disease progression (22). In the study of pretreated patients with NSCLC, the rates of hyperprogressive disease (HPD) of patients receiving PD-1/PD-L1 inhibitors and single-agent chemotherapy were 13.8 and 5.1%, respectively (23). HPD mainly occurred within two months after receiving PD-1/PD-L1 inhibitors and generally related to poor prognosis (23, 24). However, HPD in patients treated with immunotherapy plus chemotherapy was rarely reported. These differences are supported by our results. The combination therapy regimen may be able to reduce the risk of HPD related to PD-1/PD-L1 inhibitors.

In our study, the PFS of the monotherapy group was longer than that of the combination therapy group, which was different from the result of a prior retrospective analysis that compared the efficacy of a PD-1 inhibitor plus chemotherapy and/or bevacizumab and PD-1 inhibitor alone for patients with advanced NSCLC in second-line or later therapy (25). The mPFS was reported at 7.5 and 3.3 months in the combination therapy and monotherapy groups, respectively (*P* < 0.001). These differences in results from our study might be related to the KPS score at the study initiation. Their rate of KPS ≥ 90 in the combination therapy group was approximately double that in our combination therapy group (86.4 vs. 44.8%). Although the KPS score in multivariate analysis had no impact on prognosis, advanced-stage patients undergoing second-line or later therapy often had lower KPS scores. The low KPS score indicates a poor tolerance to combination therapy. Moreover, we observed that patients in the monotherapy group had a longer PFS than those in the combination therapy group in the subgroup analysis of those receiving treatment beyond second-line therapy, while there was no significant difference between the two groups in the subgroup of second-line therapy. Although the subgroup analyses did not provide adequately powered evidence for efficacy comparison, they offer a direction for future studies to determine characteristics of those patients who may benefit from combination therapy.

The efficacy of PD-1/PD-L1 inhibitors in patients with tumors harboring EGFR mutations or ALK fusions is still conflicting. A small sample study suggested that uncommon EGFR mutations in tumors may be favorable prognostic factors for therapeutic effect of nivolumab in patients with NSCLC (26). However, in a retrospective analysis of 58 patients with NSCLC treated with PD-1/PD-L1 inhibitors, the response rate was 3.6% in the EGFR mutation or ALK fusion subgroups and 23.3% in the wild-type subgroups (27). Currently, it is widely considered that PD-1/PD-L1 inhibitors have limited efficacy in patients with tumors harboring targetable mutations. A meta-analysis (28) including the CheckMate 057, KEYNOTE 010 and POPLAR trials indicated that PD-1/PD-L1 inhibitors alone did not show superior survival over docetaxel in patients with tumor EGFR mutations. Notably, in the IMMUNOTARGET study that evaluated the efficacy of PD-1/L1 inhibitors alone in patients with NSCLC with driver gene mutations, none of the twenty-three patients with ALK fusions had a disease response (29). Of the 84 patients involved in our study, 60 patients underwent somatic genetic testing. 9 of them had tumors which harbored EGFR mutations, while 2 had ALK fusions. Both patients with tumor EGFR mutations in the monotherapy group exhibited disease progression. In the combination therapy group, only one of the seven patients with tumor EGFR mutations had progressive disease, and the other six patients had stable disease. In the population with tumor ALK fusion, two patients received combination therapy, and both had progressive disease after two cycles. It is possible that patients with tumor EGFR mutations who exhibit disease progression after first-line therapy may benefit from PD-1 inhibitors plus chemotherapy. However, in the ALK fusion population, this survival benefit from PD-1/L1 inhibitors may be limited.

Lactate dehydrogenase, as a systemic inflammation indicator, has attracted attention. Multiple previous studies in various cancer types indicated that high LDH level is a poor prognostic factor for PFS or OS (30–33). A meta-analysis of 1136 patients with NSCLC treated with immune checkpoint inhibitors suggested that high LDH levels were related to shorter PFS and OS (30). Moreover, combination of dNLR and LDH levels as lung immune prognostic index (LIPI) is better at predicting these effects of PD-1/PD-L1 inhibitors (17, 18). The present study also confirmed that LDH ≤ ULN was a favorable prognostic factor for PFS in patients with pre-treated NSCLC, which was consistent with the findings of a previous study (30). Our study also suggested that male sex was a favorable prognostic factor for PFS. A meta-analysis including 20 randomized trials suggested that males benefit more than females, although the OS for both sexes can be improved by PD-1 inhibitors (34). These differences may be related to poor immunity (35, 36), high tumor mutation burden (TMB) (37) and the influence of smoking behavior on TMB in males (38). The present study’s real-world results were similar to those of previous reports and support further research in related directions, which favor selecting a population who would benefit from PD-1/PD-L1 inhibitors.

Although there was no significant difference between the two groups, the incidence of grade 3–4 AEs was relatively higher in patients receiving combination therapy. Myocarditis and pneumonitis, as the primary reasons leading to treatment interruption in the combination therapy group, are noteworthy. In our study, the incidence of grade 3–4 AEs was 7.6% in the monotherapy group and 10.2% in the combination therapy group. The incidence of any grade AEs in our study was underestimated, perhaps because low-grade AEs were not obvious for patients to actively report. A meta-analysis reported by Nishino et al. showed that the incidence of pneumonitis in NSCLC was 4.1% (39). The most common anti-PD-1/PD-L1-related fatalities were from pneumonitis in a meta-analysis predominantly consisting of NSCLC and melanoma (40). The incidence of myocarditis of any grade was only 0.06%, but the mortality of high-grade myocarditis can reach 36% in patients who received PD-1/PD-L1 inhibitors alone (41, 42). In patients receiving a combination of anti-PD-1/PD-L1 with anti-CTLA-4 inhibitors, the fatality rate can even reach 67% (42). In our study, the patient who died of pneumonitis in the combination therapy group had a history of chronic obstructive pulmonary disease, which might be a risk factor for pneumonitis. These two specific AEs should be noted in patients treated with PD-1/PD-L1 inhibitors plus chemotherapy.

In our study, patients who previously received immunotherapy were excluded. This restriction was mainly due to two reasons. First, whether it is beneficial to continue to use PD-1/PD-L1 inhibitors beyond immunotherapy progression is still unknown. Although previous studies have reported that patients with immunotherapy progression may still receive benefit from immunotherapy (43, 44), no relevant study has compared the efficacy between continuing PD-1/PD-L1 inhibitor and switching to another therapy later. The latest version of the NCCN guidelines recommend chemotherapy alone or ramucirumab plus docetaxel rather than nivolumab, pembrolizumab and atezolizumab as subsequent therapy if the disease has progressed on PD-1/PD-L1 inhibitor therapy (45). Second, previous immunotherapy potentially influences the decision to use immunotherapy combined with chemotherapy later. If disease progressed on immunotherapy, patients may prefer to receive combination therapy subsequently to allow a higher possibility of disease response. Therefore, previously treated patients would be expected to be present in the combination therapy group. To avoid this potential bias, we did not include patients who had previously received immunotherapy in this study.

As a retrospective analysis, our study has some limitations. First, the small sample size influenced the statistical power and may lead to selection bias and measurement bias. Because of the small sample size, the conclusion is not representative of the whole population. Despite adjustment by the Cox regression model, confounding factors may still exist. Further analysis with a larger sample size is necessary in the future. Moreover, a previous study has reported the rate of EGFR mutations in non-small cell lung cancer in never-smoking Asian patients to be 60.7%, while the rate of EGFR mutations in our study was only 20% (46). Selection bias may be a major reason for this difference. In the EGFR-mutated NSCLC, targeted therapy is the preferred therapeutic regimen, and immunotherapy is rarely used because of reported lower rate of benefit. Thus, patients whose tumors do not harbor EGFR mutations may preferentially be treated with immunotherapy, which may explain the lower rates of somatic EGFR mutations in our study. Second, the available baseline features in our retrospective study were limited. Some important clinical information, which may affect the survival time and treatment response, such as PD-L1 tumor proportion score or TMB level and baseline lung function, was not available for all patients.

## Conclusion

In conclusion, our study suggested that the addition of chemotherapy to PD-1 inhibitors did not improve clinical outcomes compared to monotherapy with PD-1/PD-L1 inhibitors in second-line or later treatment settings for patients with advanced NSCLC. Our observations provide directions for future treatment studies and valuable clues about prognostic factors and adverse events with use of PD-1/PD-L1 inhibitors with or without chemotherapy in this patient population.

## Data Availability Statement

The datasets generated for this study are available on request to the corresponding author.

## Ethics Statement

The studies involving human participants were reviewed and approved by the Ethics Committee of Shandong Cancer Hospital Affiliated to Shandong First Medical University. The patients/participants provided their written informed consent to participate in this study.

## Author Contributions

HZ designed the study. XZ collected the data of clinical trials together with SX and MW and drafted the manuscript. XJ, JL, and YT coordinated. All authors read and approved the final manuscript.

## Conflict of Interest

The authors declare that the research was conducted in the absence of any commercial or financial relationships that could be construed as a potential conflict of interest.

## Abbreviations

AEs: adverse events
ALK: anaplastic lymphoma kinase
CTCAE: Common Terminology Criteria for Adverse Events
CTL: cytotoxic T lymphocyte
DCR: disease control rate
dNLR: derived neutrophil to lymphocyte ratio
EGFR: epidermal growth factor receptor
HPD: hyperprogressive disease
HR: hazard ratio
KPS: Karnofsky performance status
LDH: lactate dehydrogenase
NSCLC: non-small-cell lung cancer
OS: overall survival
PD-1: programmed death-1
PD-L1: programmed death-ligand 1
PFS: progression-free survival
RECIST: Response Evaluation Criteria in Solid Tumors
SD: stable disease
TKI: tyrosine kinase inhibitor
TPS: tumor proportion score
ULN: upper limit of normal.
